# Comparison of insertion/deletion calling algorithms on human next-generation sequencing data

**DOI:** 10.1186/1756-0500-7-864

**Published:** 2014-12-01

**Authors:** Dalia H Ghoneim, Jason R Myers, Emily Tuttle, Alex R Paciorkowski

**Affiliations:** Center for Neural Development and Disease, University of Rochester Medical Center, 601 Elmwood Avenue, Rochester, NY USA; Genomics Research Center, University of Rochester Medical Center, Rochester, NY USA; Departments of Neurology, Pediatrics, and Biomedical Genetics, University of Rochester Medical Center, Rochester, NY USA

**Keywords:** Next generation sequencing, Indels, Validation, Concordance, GATK, Pindel

## Abstract

**Background:**

Insertions/deletions (indels) are the second most common type of genomic variant and the most common type of structural variant. Identification of indels in next generation sequencing data is a challenge, and algorithms commonly used for indel detection have not been compared on a research cohort of human subject genomic data. Guidelines for the optimal detection of biologically significant indels are limited. We analyzed three sets of human next generation sequencing data (48 samples of a 200 gene target exon sequencing, 45 samples of whole exome sequencing, and 2 samples of whole genome sequencing) using three algorithms for indel detection (Pindel, Genome Analysis Tool Kit's UnifiedGenotyper and HaplotypeCaller).

**Results:**

We observed variation in indel calls across the three algorithms. The intersection of the three tools comprised only 5.70% of targeted exon, 19.52% of whole exome, and 14.25% of whole genome indel calls. The majority of the discordant indels were of lower read depth and likely to be false positives. When software parameters were kept consistent across the three targets, HaplotypeCaller produced the most reliable results. Pindel results did not validate well without adjustments to parameters to account for varied read depth and number of samples per run. Adjustments to Pindel's M (minimum support for event) parameter improved both concordance and validation rates. Pindel was able to identify large deletions that surpassed the length capabilities of the GATK algorithms.

**Conclusions:**

Despite the observed variability in indel identification, we discerned strengths among the individual algorithms on specific data sets. This allowed us to suggest best practices for indel calling. Pindel's low validation rate of indel calls made in targeted exon sequencing suggests that HaplotypeCaller is better suited for short indels and multi-sample runs in targets with very high read depth. Pindel allows for optimization of minimum support for events and is best used for detection of larger indels at lower read depths.

**Electronic supplementary material:**

The online version of this article (doi:10.1186/1756-0500-7-864) contains supplementary material, which is available to authorized users.

## Background

Indels are the second most common type of genomic variant and the most common type of structural variant [1] with an expected ~1.6 million collective indel polymorphisms in the human population [2]. Between 0.13-0.4 million short indels are found per individual [3, 4], and of these, 192–280 are frameshift (1000 Genomes Project Consortium et al., 2010). The presence of indels can be associated with disease when they disrupt the amino acid sequence of coding regions or the regulatory functions of non-coding regions [5–8]. Next generation sequencing (NGS) is a common and cost-effective method for identifying variation in the human genome. However, identification of indels can prove more challenging than identification of single nucleotide variants (SNVs) [1, 9] especially with the presence of single nucleotide polymorphisms (SNPs), sequencing errors, and polymerase chain reaction (PCR) enrichment bias [10]. While the majority of indels are 1–10 bp long [1, 11], known indels that range up to 10,000 base pairs are present in the human genome [12]. Detection of longer indels is especially challenging due to NGS short read length, making it difficult to identify indels larger than the read length. Few studies have examined the indel calling capabilities of available tools on multiple samples of human data. We have run a comparison of commonly used indel detection tools: Pindel, the Genome Analysis Toolkit's (GATK) UnifiedGenotyper, and GATK's HaplotypeCaller on a diverse set of targets in human NGS data.

GATK’s UnifiedGenotyper is a naive Bayesian genotyper that uses pileup of sequence reads to determine the posterior probability of each genotype. UnifiedGenotyper is successful at calling SNVs, identifying variants on chromosome 1 with 99.76% concordance to sites in dbSNP and a 99.84% concordance figure with HapMap sites [13]. However, studies specific to indel calling are limited. One study reported a 92% validation rate using UnifiedGenotyper for indel detection in human whole exome data [14]. Another study using simulated short indels reported high positive predictive value for UnifiedGenotyper, but decreased sensitivity at lower read depths (< 10x) [15].

A newer variant-calling tool, HaplotypeCaller, was introduced in GATK version 2.0. HaplotypeCaller detects variants using a combination of *de novo* assembly and a Hidden Markov Model. Literature regarding the performance of HaplotypeCaller is limited. A study of GATK's variant calling capabilities using a previous version of the tool (2.2-2) reported lower validation of indels called by HaplotypeCaller (55.9%) in comparison to UnifiedGenotyper (92.0%) [14]. This tool is under continuous development, and a more recent version (2.6-4) was included in our comparison.

Pindel is a tool capable of identifying indels as well as other structural variants in paired-end read data. Pindel’s pattern-matching algorithm determines break points using mate-pair reads where one end is mapped and the other is unmapped. This is followed by reconstruction of a complete read at the breakpoints to predict the presence of indels. On simulated data, Pindel identified up to 80% of deletions ranging from 1–16 bp in size with less than 2% false negative rate. Insertions were also detected at a rate of approximately 80% [16].

The performance of many currently available indel calling tools has been compared primarily using simulated data [15, 17, 18]. These studies are informative regarding the false positive rate and sensitivity of these tools, but few studies have reported the performance of these tools on a research cohort of human sequencing data. We compared indel calling capabilities on human target capture of 200 genes, whole exome sequence data, and whole genome sequence data using Pindel, UnifiedGenotyper and HaplotypeCaller. The quantity, size, read depth and other characteristics of called indels specific to each program were compared, and the concordance of the indels called across the various programs was determined. This study can be useful to researchers when selecting an appropriate tool for their specific needs. We clarify the overall ability of currently available indel callers to detect these genomic variations in real human subject data, and suggest best practices for the identification of indels in human next generation sequencing data.

## Results

### Sequencing

We achieved mean read depth of 639x for the TES samples, 74x for the WES, and 24x for the WGS samples.

### Characteristics of indels called

**Figure 1:**
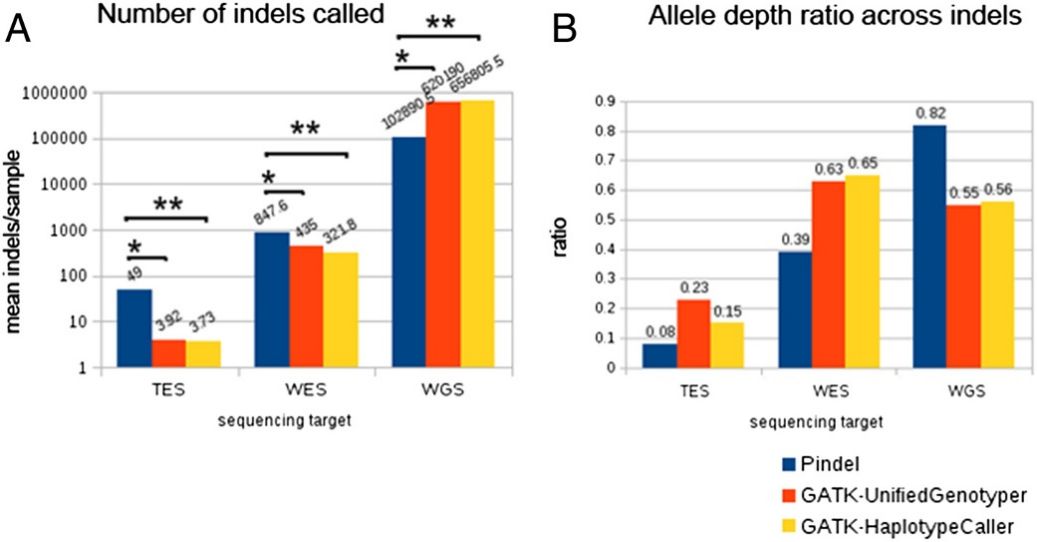
**Performance characteristics of three indel calling algorithms applied to three human targets. (A)** Comparison of number of indels called. **(B)** Comparison of allele depth ratio across indels called. Pindel called more indels in TES and WES samples, but less indels in the WGS samples than the GATK algorithms.

Pindel made significantly more (p < 0.0005) indel calls in the TES and the WES samples than either of the GATK tools. Pindel called 49 indels per sample in the TES and 847.6 indels per sample in the WES compared to 3.92 and 3.73 indel calls per sample in the TES and 435 and 321 indel calls per sample in the WES data made by UnifiedGenotyper and HaplotypeCaller respectively. In contrast, Pindel called significantly fewer (p = 0.013) indels than either of the GATK tools in the WGS samples with the settings we used throughout the three sequencing datasets. These findings are summarized in Figure 1A. When Pindel was run with -M reduced to 10, the number of calls made by Pindel increased to 574,892 indel calls per sample compared to UnifiedGenotyper's 620,190 indel calls and HaplotypeCaller's 656805.5 calls. With -M set to 3, Pindel called 680785.5 indels, more than the other two tools (Additional file 1: Figure S1).

The read depth over indels called by UnifiedGenotyper was consistently higher than read depth over indels called by HaplotypeCaller especially in the TES and WES runs. Both GATK algorithms had higher mean allele depth in support of the called variants than Pindel for both the TES and WES samples. However, in the WGS samples, Pindel called variants that had a higher allele depth than either of the other algorithms (Figure 1B).

The mean indel size in TES and WES sequencing sets was higher in Pindel (90.1 bp in TES and 317.1 bp in WES) compared to both UnifiedGenotyper (7.6 bp in TES and 4.0 bp in WES) and HaplotypeCaller (7.4 bp in TES and 4.0 bp in WES). Pindel called 47 unique large deletions (> 2000 bp) in the 45 WES samples. The largest of these was 30,861 bp long (just below the maximum size threshold parameter set for Pindel) compared to maximum deletion size of 59 bp by UnifiedGenotyper and 113 bp by HaplotypeCaller. HaplotypeCaller was capable of calling the largest insertions (up to 108 bp long) followed by UnifiedGenotyper (59 bp) and Pindel (57 bp). Indels called by each algorithm in the TES and WES data had median size in the range of 1–6.7 bp. The only significant difference in median size was in Pindel TES samples compared to the GATK algorithms (p < 0.0005).

### Concordance

**Figure 2:**
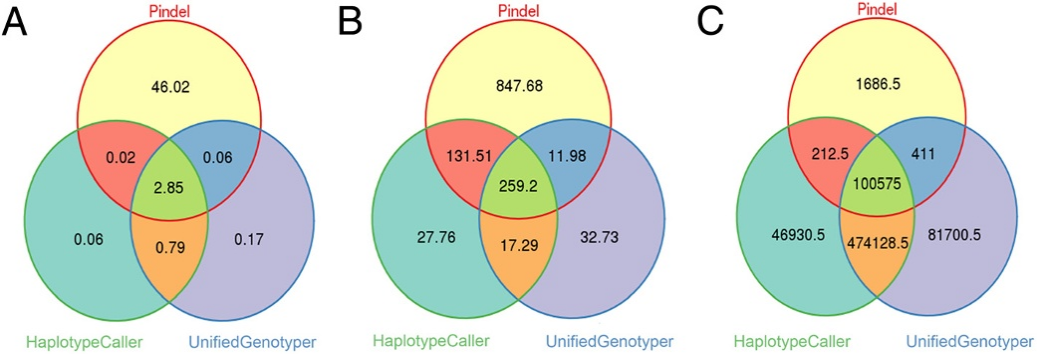
**Concordance of indels called by Pindel, GATK-HC and GATK-UG in targeted exon capture (A), whole exome sequencing (B), and whole genome sequencing (C) data sets without adjustment to software parameters.** Concordance was low in the intersection of the three tools across all targets. UnifiedGenotyper and HaplotypeCaller had higher concordance of calls, but did not detect many of the indels called by Pindel in TES and WES.

The concordance rate for indels called by the three algorithms was only 5.70% in TES, 19.52% in WES, and 14.25% in WGS (Figure 2). Indels detected by Pindel in the TES data had the highest discordance with other algorithms. Only 5.99% of TES indels and 32.21% of WES indels detected by Pindel were called by either of the GATK tools. In contrast, 98.36% of WGS indels called by Pindel at M = 30 were also identified by at least one of the GATK tools. The GATK algorithms produced highly concordant results in TES (92.15%) and WGS (81.64%). The concordance between UnifiedGenotyper and HaplotypeCaller in WES was not as high (57.55%). Running Pindel WGS samples with lower values for -M resulted in increasing concordance. There was 24.37% concordance in the three algorithms when -M was set to 10 supporting reads, and 25.25% at the default setting of 3 supporting reads.

Discordant indels tended to have lower mean read depth than the mean read depth across all indels. The exceptions to these were Pindel WGS calls (with M = 30) where the unique indels called had a mean allele depth of 47.3 reads supporting variant alleles compared to a mean depth of 19.9 supporting reads across all indels called by Pindel. Pindel called a higher number of discordant indels per sample in the TES and WES than UnifiedGenotyper or HaplotypeCaller. A percentage of these discordant indels (4.00% in TES and 2.99% in WES ≥ 2000 bp) were large deletions that were not captured by either GATK algorithm. We found that with consistent parameter settings, Pindel called more indels in targets with high read depth, but of these only 28% in the TES and 21% in the WES validated.

### Effects of multi-sample runs

**Figure 3:**
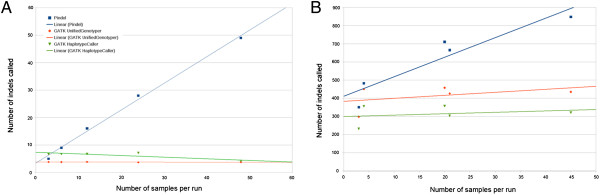
**Variation in number of indel calls per sample depending on the number of samples analyzed simultaneously in targeted exon capture (A) and whole exome sequencing (B) data.** Pindel showed a dramatic increase in indel calls as more samples were added to analysis. UnifiedGenotyper and HaplotypeCaller also showed variance in the number of indel calls depending on the number of samples analyzed simultaneously. However, there was not necessarily a positive correlation.

In our TES data, increasing the number of samples run simultaneously resulted in a significant increase in the number of calls made by Pindel (p < 0.00005 comparing results from 3 versus 48 samples) at a rate of 0.97 indel calls with each additional sample (Figure 3A). HaplotypeCaller showed a significant decrease in the number of calls per sample with the addition of more samples (p < 0.00001 with 3 versus 48 samples) but at a slower rate than Pindel. UnifiedGenotyper did not show any significant changes in the number of calls. When only three WES samples were analyzed simultaneously, Pindel still made a significantly higher number of calls than both UnifiedGenotyper (p = 0.002) and HaplotypeCaller (p = 1.199e-7).

All three algorithms applied to the WES data showed a positive correlation between the number of samples analyzed simultaneously and the number of indel calls (Figure 3B) and had an increase in the number of indel calls in 3-sample runs compared to 48-sample runs. Pindel was most affected by the addition of samples, increasing at a rate of 10.72 indels with each additional sample added to the analysis. The GATK algorithms also showed an increase in indel calls as the number of samples increased. This increase was smaller, with 1.66 indels per additional sample in UnifiedGenotyper and 0.78 indels per additional sample in HaplotypeCaller. In all of the WES runs tested, Pindel called more variants than either GATK tool regardless of the number of samples analyzed simultaneously.

### Validation of calls made depending on target size

**Figure 4:**
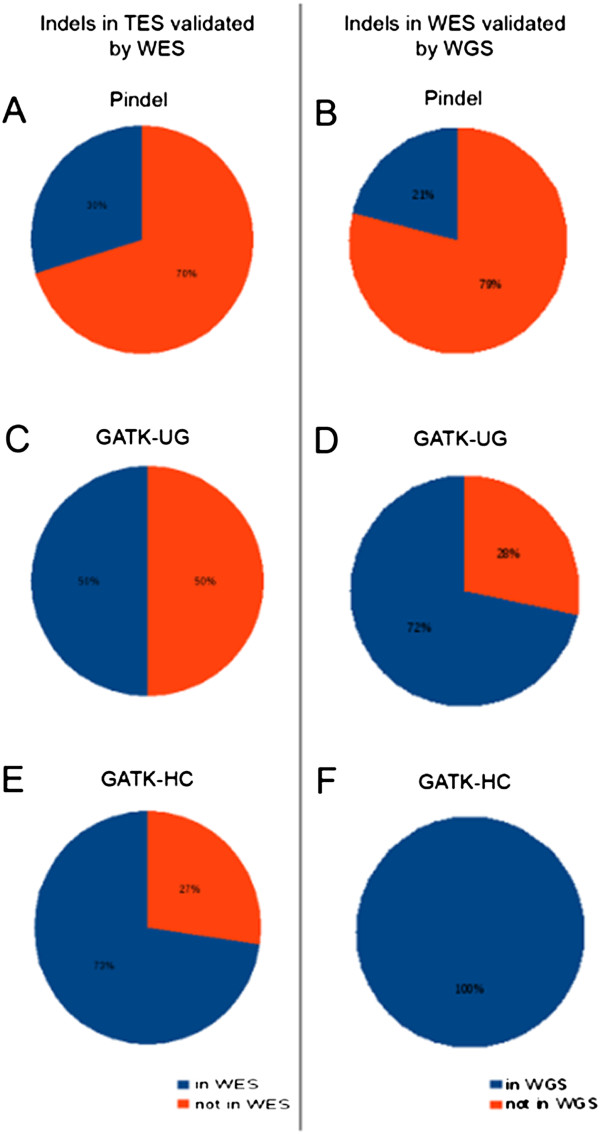
**Validation of indels called by Pindel (A,B), GATK-UG (C,D), and GATK-HC (E, F) in targeted exon sequencing with whole exome sequencing, and whole exome sequencing with whole genome sequencing using uniform parameter settings across the targets.** HaplotypeCaller performed the most reliably across the different targets. Pindel indel calls had the lowest validation rates when settings were unchanged across the targets. Lower TES validation rates in all three tools suggest either more false positives due to high depth of coverage in TES or insufficient depth or coverage in WES to validate the TES calls.

Only 30% of TES indels identified by Pindel were also called in Pindel's corresponding WES data. A larger percentage (50%) of TES indels called by UnifiedGenotyper and 73% of the TES indels called by HaplotypeCaller were found in the corresponding WES runs. In our WES data, only 21% of Pindel's WES indels were found in WGS data. UnifiedGenotyper identified 72% of the WES indels in WGS, and 100% of the WES indels identified by HaplotypeCaller were also identified in the WGS samples. Validation data are summarized in Figure 4.

### Validation results with varied Pindel M values

**Figure 5:**
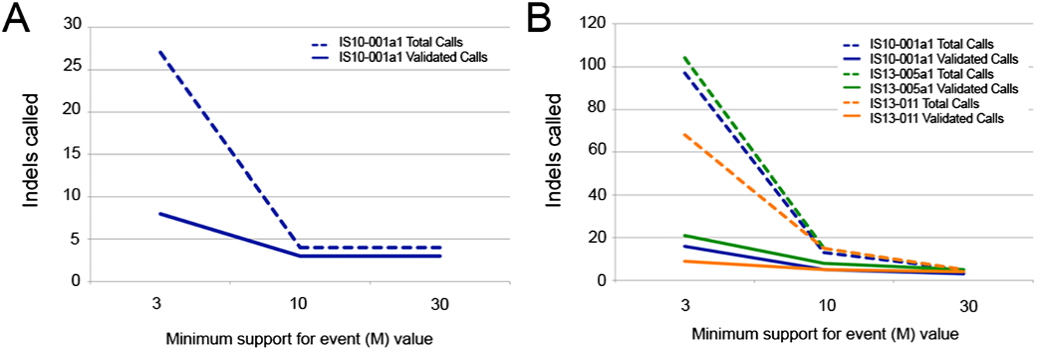
**Comparison of Pindel results when minimum support for event (M) value is adjusted in one sample TES analysis (A) and three-sample TES analysis (B).** Adjusting the M parameter in Pindel improved validation rates. When one TES sample was run, adjusting M to 10 increased the validation rate to 75%. Increasing M to 30 did not have an effect on the validation rate. When three samples were run simultaneously, adjusting M to 30 produced results with the highest validation rate. Adjustments to M must take into account the number of samples as well as the depth of coverage of the target.

Varying the minimum support for event (M) value upon which Pindel bases its calls resulted in differing numbers of indels validated across the targets. Analysis of a single TES sample using Pindel with M = 10 increased validation to 75%, and there was no benefit gained in validation with an increase to M = 30 (Figure 5A). When we analyzed three TES samples with Pindel, increasing the M value to 30 resulted in validation rates from 60-100% across the three samples (Figure 5B). Interpretation of our WES and WGS sample validation should be cautious, as we only had one WGS sample with corresponding WES data, but in that sample varying M values improved validation from 21% with M = 30 to 78% with M = 3 (Additional file 2: Figure S2).

### Large deletions called by Pindel

Looking at the WES large deletions (>2000 bp) called by Pindel, we found that out of 46 unique deletions, 45 were detected in multiple samples. On average, each deletion was found in 24.78 of the 45 samples. As we had trio WES data, we found that all large deletions found in probands were inherited from a parent, and none were *de novo*. Frameshift deletions comprised 65.22% of the deletions called. Using UCSC’s RepeatMasker we found that all 46 deleted regions overlapped with known genomic areas of repeats. Every deletion also overlapped with copy number variants found in the DGV database of known structural variants. In the subset of 12 large deletions in sample DB13-001, exon one of the protocadherin alpha gene cluster (PCDHA8,9,10) was found to map to other genomic regions with sequence similarity.

## Discussion

The analysis of insertions and deletions continues to be a challenging area in analysis of next-generation sequencing data. There is variability in the approach taken by current algorithms, with GATK analyzing insertions and deletions simultaneously, while Pindel performs this analysis separately. We observed variability across all three indel calling algorithms when applied to a variety of real human next-generation sequencing data sets. Others have reported lack of concordance in indel calling tools when applied to simulated data [15, 17, 18] and human WES data [14]. Both GATK algorithms produced highly concordant results in TES and WGS, but did not perform as well when applied to WES data. Therefore, we concur with the caution voiced in the literature regarding the high false positive rate of indel calling in next-generation sequencing data [14, 19, 20].

Overall, we found that GATK’s HaplotypeCaller had the highest concordance and validation. We recommend that researchers use concordance between HaplotypeCaller and Pindel with supporting read adjustment to optimally call indels that validate. We observed the greatest variability when these algorithms were applied to smaller NGS targets. GATK’s HaplotypeCaller provided the most consistently validated indel calls across all target sizes. In contrast, Pindel called a greater number of indels that were not validated by the other algorithms when applied to the same data obtained from the same sequencer. However, Pindel’s calls from our WGS data were well validated.

Pindel made a significantly higher number of indel calls per sample than the GATK tools except in the WGS samples where the number of calls made by Pindel was significantly lower. This was true even with adjustments to Pindel's settings for higher stringency. Only 30% of calls in the TES and 21% in the WES validated, indicating more of these calls made in targets with high read depth were false positives. Our findings are consistent with a comparison of indel calling algorithms on simulated human chromosome 22 data suggesting that Pindel had higher false positive and false negative rates for indels 1–5 bp long while GATK had higher false-positive and false-negative rates for indels 30–100 bp long at read depth > = 20x [17]. As 75.36% of our called indels were in the 1–5 bp range, it is possible that the high number of calls made by Pindel in the TES and WES could be the result of more false positives in the small indel category.

The larger mean indel size called by Pindel was likely due to the identification of very large (>2000 bp) deletions. Neither GATK algorithm is capable of identifying these large deletions [17]. In the case of large insertions, Pindel and GATK UnifiedGenotyper both identified insertions just under 60 bp in length. In our data, HaplotypeCaller identified the largest insertions out of the three algorithms, and these did validate in WGS data. Indels in our TES and WES analyses had median sizes within the range of 1–6.7 bp, consistent with previous findings that the majority of short indels (<60 bp) are less than 10 bp in length [11]. Pindel had a lower median indel size than the GATK tools in the TES and WES data, and this difference was significant in the TES run (p < 2.2e-16). This is further evidence that Pindel may have called more small (1–5 bp) false positive indels than GATK at very high read depth while GATK may have called more false positive large indels than Pindel.

We also observed an interesting behavior by Pindel where more indels were called in the TES and WES data when multiple samples were analyzed simultaneously. This may be because Pindel establishes calls in part based upon the number of supporting reads. Running only two samples together may have contributed to the low number of indel calls made by Pindel in our WGS data. However, even when only three WES samples were analyzed simultaneously, Pindel still made a significantly higher number of calls than both UnifiedGenotyper (p = 0.002) and HaplotypeCaller (p = 1.199e-7), and when three TES samples were analyzed together, the number of indel calls made by Pindel was within the range of the number of calls made by UnifiedGenotyper and HaplotypeCaller. This suggests that the number of samples is not the only factor contributing to the lower number of calls. The lower coverage in the WGS data along with the settings used, especially the number of supporting reads (−M) is likely to be the cause. We conclude that with varying read depth, the value of -M has an effect on the number of calls made and must be adjusted accordingly. This can be problematic when searching for indel calls found in only one sample in a multi-sample run with lower coverage. Setting -M too high will not detect low-coverage indels, while setting it too low will call many indels in common among the samples. The solution is to set M to a lower value and filter out these variants that are common to many samples.

GATK HaplotypeCaller and UnifiedGenotyper showed less variation in number of indels called when more samples were added to analysis, and in the case of our TES data, there was not necessarily a positive correlation between the number of indels called and the number of samples analyzed. This variation in the number of calls may reflect GATK's ability to identify indels more accurately with added samples. GATK best practices suggest running UnifiedGenotyper and HaplotypeCaller on 30 or more samples, as this increases the statistical power and improves accuracy of variant calls [21].

Concordance across the three tools was low in all of our targets. GATK-UnifiedGenotyper and GATK-HaplotypeCaller produced calls with higher concordance in TES and WGS than previously reported in earlier versions of HaplotypeCaller (54.1% in version 2.2-2 and 70.7% in version 2.4-9) [14]. Low concordance in TES and WES in the intersection of the three tools is due to a high number of indel calls by Pindel that were not detected by the GATK algorithms. In the case of WGS, the high -M setting resulted in relatively few indel calls by Pindel in comparison to the GATK tools. 98% of these indels were also identified by the other tools and likely to be high confidence calls. However, the low number of calls made by Pindel in WGS data resulted in lower concordance among the three tools. The level of concordance in WGS samples improved with adjustment to Pindel's parameters and was highest for the WGS samples when Pindel was run at the default value for M (3).

Validation of indels across their corresponding samples in different targets varied among the three tools. The validation rate in all three tools was lower from TES to WES than WES to WGS. HaplotypeCaller had the most reliable results when applied to the various targets. Previous studies on indel callers have found that the sensitivity of variant callers is positively correlated with read depth of the sequenced regions [15, 17, 22]. In combination with the increase in false positives that accompanies increases in read depth, it is expected that some calls in the high read-depth targets will not be found in targets with lower read-depth. Our findings suggest that Pindel may be more affected by low or high read depth than the GATK algorithms, either due to a higher rate of false positives when depth is high, lower sensitivity at the lower read depth, or a combination of the two. The low validation rate of Pindel's WES sample in WGS data can be attributed to the low number of indels detected in WGS due to lower depth of coverage coupled with the stringent M parameter setting. Pindel's validation rate in WES exceeded that of UnifiedGenotyper with adjustments to M in the WGS run. We did not make adjustments to UnifiedGenotyper or HaplotypeCaller to maximize validation.

Adjustment of Pindel’s minimum support for event value (M) greatly improved both concordance and validation of indels called in our TES samples. In our analysis of a single TES sample, validation improved with M = 10 compared to M = 3, but there was no benefit to analysis with M = 30. In contrast, analysis of 3 TES samples simultaneously had higher validation rates when M was set to 30 compared to M = 10 or M = 3. From this, we conclude that for researchers analyzing smaller target capture sequencing with higher read depth, varying M values will improve the validation of indels called.

When analyzing smaller targets with higher read depth, researchers should keep in mind the possibility of more false positive calls made by indel-calling software in these targets. The percentage of TES indels that are not identified in the WES runs by all three algorithms also raises questions about the ability of current sequencing technology to achieve sufficient read depth over large genomic regions to capture all indels successfully. This is a paradox, since large indels spanning genomic regions may not be visible by smaller target captures, yet read-depth in WES and WGS may not be sufficient to allow current software (including those tested) to identify these indels with confidence.

### Best practices recommendations

Our comparison of multiple indel calling algorithms across targets of varying size in human subject data allows us to make several best practices recommendations to researchers undertaking similar analyses (Table 1). We recommend the use of HaplotypeCaller to detect small indels less than 10 bp long. For indels in the 10 bp-100 bp range, HaplotypeCaller in combination with Pindel can be used for indel detection with higher confidence. It is important to adjust the number of supporting reads for Pindel to suit the target size and the number of samples in the run. Default settings are optimal for WGS data or other sequencing data sets with relatively lower read depth. Multi-sample TES or WES runs with higher read depth require adjustment of M to at least 10. Pindel may not be optimal for detection of low read depth indels that occur in a single sample in a multi sample run. To detect these elusive indels, Pindel can be run with a low value for M, and results can then be filtered. Pindel is the only tool of the three capable of detecting larger deletions. Pindel may also be the tool of choice for datasets that require higher sensitivity at the potential expense of concordance and validation. If researchers are aware of the strengths and weaknesses among the three algorithms tested and adjust for them accordingly, optimal results in terms of concordance and validation will be obtained. There is continuous development of new tools for indel and structural variant detection, including Platypus [23], a new variant-calling tool developed for use on whole exome sequence and whole genome sequence data. Further testing will be required to determine the strengths and weaknesses of the most recent tools.

**Table 1:** **Best practices for optimal identification of indels in human next-generation sequencing data**

Target	Small (1–10 bp) indels	Mid-sized (10–100 bp) indels	Large (>2000 bp) deletions**
Human 200 gene TES capture (1.5-1.6 Mb) with read depth ~600x	GATK-HC	Run both GATK-HC and Pindel with adjustments to parameters*.	Pindel
	Validate with Pindel helpful if analyzing <6 samples		
Human WES capture (65–75 Mb) with read depth ~100x	GATK-HC	Run both GATK-HC and Pindel with adjustments to parameters*.	Pindel
	Validate with Pindel helpful if analyzing <6 samples		
Human WGS capture (3.2 Mb) with read depth ~30x	GATK-HC	Run both GATK-HC and Pindel with M = 3.	Pindel
	Validation with Pindel may be helpful, but run with M = 3		

*Adjust Pindel M value to 10 if analyzing single sample; adjust M to 30 if analyzing multiple samples.

**Pindel does not identify large insertions, but can identify the breakpoints for these insertions. These were not included in this analysis.

## Conclusions

Variability in indel calls was high across the three indel detection tools tested. Understanding the strengths of each tool can improve identification of indels in sequencing data. GATK’s HaplotypeCaller had the best validation of indels found across different sequencing targets. While GATK UnifiedGenotyper and HaplotypeCaller are both well suited for very small indels, HaplotypeCaller identifies more indels in low depth of coverage than UnifiedGenotyper. Pindel has the ability to detect larger deletions as well as break points for larger insertions that are not identifiable by either of the GATK algorithms. However, Pindel was the most sensitive to skewing of results depending on the read-depth of the target and the number of samples analyzed simultaneously. Adjusting Pindel’s minimum support for event (M) value depending on the number of samples analyzed greatly improved the validation of indels called and illustrates the importance of adjusting software parameters to suit the read depth and coverage of datasets. This is the first comparative analysis of indel calling algorithms on a research cohort of human next generation sequencing data.

## Methods

### Human subject ascertainment

All human subjects sequenced underwent informed consent as part of protocols approved by the Research Subjects Review Board of the University of Rochester Medical Center.

### Sequencing

Target capture of 200 genes using the Agilent SureSelect Custom 2.9 Mb capture kit was performed on 48 human DNA samples (a combination of blood and saliva derived). Whole exome capture of 45 saliva-derived human DNA samples was performed with the Agilent SureSelect V5 + UTR capture kit. Whole genome capture was performed on two saliva-derived human DNA samples using the Illumina TruSeq DNA PCR-free kit. All paired-end sequencing was performed on the same Illumina HiSeq2500.

### Analysis pipeline

Demultiplexing and cleaning of raw reads were performed with bcltofastq-1.8.4, seqclean-x86_64 l, fastqc-0.10.1, and fastx_toolkit_0.0.13. The sequencing reads were mapped with bwa-0.6.2 to the hg19 reference genome and were then sorted and converted to bam with samtools-0.1.17. In preparation for variant-calling, the reads were soft-clipped and duplicates marked using Picard-1.84. Gatk −2.3-9 was used for IndelRealignment and base quality score recalibration according to GATK best-practices [21]. The resulting bam files were then run through Pindel, GATK UnifiedGenotyper, and GATK HaplotypeCaller for indel calling. Each program was run in a multi-sample run containing all 45 samples for whole exomes, 48 samples for the 200 gene targeted capture, and 2 samples for the whole genome sequencing. Each tool was run using identical parameter settings across the three targets.

UnifiedGenotyper was run with default settings as documented in GATK Best Practices, except the confidence (phred-scaled) threshold for variants emission was lowered to 10.0 (default stand_emit_conf is 30.0) and the coverage was down-sampled to 1000 per sample (default d_cov is 250). The target list (−L) option was removed for variant calling in the WGS samples. GATK HaplotypeCaller was run using a newer version of GATK (2.6-4) with similar parameters to UnifiedGenotyper. Once again, the -L target list option was omitted for variant calling in the WGS samples.

Pindel was also run using default settings except for the minimum supporting reads (−M) across samples were increased to 30 and the minimum mismatches to map candidate reads to the reference was increased to three to control the large number of indels called by Pindel. To adjust for low calls made by Pindel in the WGS due to these stringent setings, WGS samples were also rerun through Pindel with -M set to 3 and 10. Pindel's short insertions(_SI) and deletions(_D) output were used in our analysis. Pindel output was converted to vcf using pindel2vcf (included in Pindel distribution) and annotated using Annovar. Command line parameters for GATK UnifiedGenotyper, HaplotypeCaller, and Pindel are listed in Additional file 3: Table S1.

### Characteristics of indels called

In order to characterize the types of indels that were identified by each tool, indel size, allele and read depth, proximity to known segmental duplications, as well as whether the indels were frameshift or nonframeshift indels were observed.

### Concordance of calls

Concordance of the indel calls from the three tools was determined by the number of identical calls made in each sample. Two calls were determined to be identical if they had the same start and end positions +/− 10 bp. The predicted content of the called indels was not used to determine concordance.

### Effect of multiple-sample runs

To determine the effect of the number of samples per run on indel calling capabilities, TES samples were run through each of the three tools with 3, 6, 12, 24, and 48 samples per run, and WES samples were run with 3–4 samples per run, 20–21 samples per run, and 45 samples per run. The number of indels called per sample for each run was compared.

### Validation of indel calls

Indel calls from 3 TES samples were validated by comparing to the corresponding WES data for those samples. Similary, indels called in one WES sample were validated through calls in the WGS data for that sample. The three TES samples and their corresponding WES samples were also run through Pindel in 3-sample runs using various -M values (minimum reads supporting an event) to determine the effect of this parameter on validation rates. The values tested were 3, 10, and 30. The single WES sample and its corresponding TES and WGS samples were run through Pindel as single-sample runs using the three different values for –M. Validation rates were compared.

### Large deletions called by Pindel

Large Deletions (> 2000 bp) called by Pindel in the WES data were evaluated using UCSC’s RepeatMasker and the Database of Genomic Variants (DGV) to determine if these calls contained domains with known repeats or duplications. Vista was used to identify overlap with coding and/or conserved regions of the human genome. In a subset of 12 large deletions in WES sample DB13-001, Blastn of the 50 bp flanking regions was used to determine if mapping to multiple genomic regions occurred.

## Availability of supporting data

The data sets supporting the results of this article are available at https://paciorkowski-lab.urmc.rochester.edu/data.

### URLs cited

BWA http://bio-bwa.sourceforge.net/.

Picard http://picard.sourceforge.net/.

SAMtools http://samtools.sourceforge.net/.

GATK https://www.broadinstitute.org/gatk/.

Pindel http://gmt.genome.wustl.edu/pindel/current/.

Annovar http://www.openbioinformatics.org/annovar/.

Vista http://genome.lbl.gov/vista/index.shtml.

Database of Genomic Variants http://dgv.tcag.ca/dgv/app/home.

UCSC http://genome.ucsc.edu/index.html.

## Electronic supplementary material

Additional file 1: Figure S1: Comparison of number of indels in whole genome sequencing data called by Pindel at varying minimum support for event (M) values, GATK UnifiedGenotyper, and GATK HaplotypeCaller. (PNG 17 KB)

Additional file 2: Figure S2: Validation of indels called by Pindel in whole exome sequencing was optimal at a minimum support for event (M) value of 3, compared to M = 10 and M = 30. (PNG 17 KB)

Additional file 3: Table S1: Command line parameters for GATK UnifiedGenotyper, HaplotypeCaller, and Pindel used in this study. (DOC 27 KB)
