# Supplementary figures and images for: Comparison of insertion/deletion calling algorithms on human next-generation sequencing data
Source: BMC Res Notes. 2014 Dec 1;7:864. doi: 10.1186/1756-0500-7-864 (PMC4265454; doi:10.1186/1756-0500-7-864)

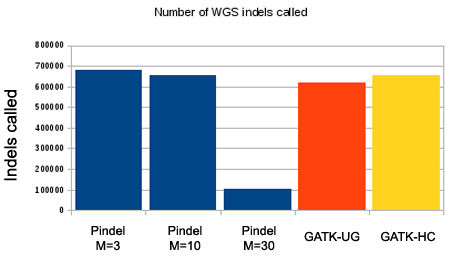

Supplement: Supplementary file 1 — Additional file 1: Figure S1: Comparison of number of indels in whole genome sequencing data called by Pindel at varying minimum support for event (M) values, GATK UnifiedGenotyper, and GATK HaplotypeCaller. (PNG 17 KB) [file 13104_2014_3378_MOESM1_ESM.png]

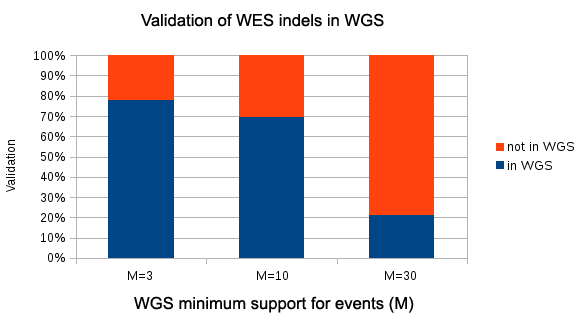

Supplement: Supplementary file 2 — Additional file 2: Figure S2: Validation of indels called by Pindel in whole exome sequencing was optimal at a minimum support for event (M) value of 3, compared to M = 10 and M = 30. (PNG 17 KB) [file 13104_2014_3378_MOESM2_ESM.png]
