# Supplementary material for: Comparison of insertion/deletion calling algorithms on human next-generation sequencing data
Source: BMC Res Notes. 2014 Dec 1;7:864. doi: 10.1186/1756-0500-7-864 (PMC4265454; doi:10.1186/1756-0500-7-864)
Supplement: Supplementary file 3 — Additional file 3: Table S1: Command line parameters for GATK UnifiedGenotyper, HaplotypeCaller, and Pindel used in this study. (DOC 27 KB) [file 13104_2014_3378_MOESM3_ESM.doc]

**Additional file 3: Table S1:** Command line parameters for GATK UnifiedGenotyper, HaplotypeCaller, and Pindel used in this study.

| java -jar GenomeAnalysisTK.jar -R hg19.fasta -T UnifiedGenotyper -glm INDEL -L <sequencing_target_list.bed> -I <sample.bam> -o <output.vcf> -stand_call_conf 30.0 -stand_emit_conf 10.0 -dcov 1000 |
| --- |
| java -jar GenomeAnalysisTK.jar -R hg19.fasta -T HaplotypeCaller-L <sequencing_target_list.bed>-I <sample.bam> -o <output.vcf> -stand_call_conf 30.0 -stand_emit_conf 10.0 -minPruning 4 |
| pindel -f hg19.fasta -i <sample.bam> -c ALL -M 30 -a 3 -o <output_file> |
